# The Effect of Reactive Sputtering on the Microstructure of Parylene-C

**DOI:** 10.3390/ma15155203

**Published:** 2022-07-27

**Authors:** Akeem Raji, Ye-Seul Lee, Seung-Yo Baek, Ji-Hyeon Yoon, Akpeko Gasonoo, Jonghee Lee, Jae-Hyun Lee

**Affiliations:** 1 Department of Creative Convergence Engineering, Hanbat National University, 125 Dongseo-daero, Yuseong-gu, Daejeon 34158, Korea; akeemraj61@gmail.com (A.R.); koko_42@naver.com (Y.-S.L.); hyo08177@naver.com (S.-Y.B.); jason7928@naver.com (J.-H.Y.); jonghee.lee@hanbat.ac.kr (J.L.); 2 Research Institute of Printed Electronics & 3D Printing, Hanbat National University, 125 Dongseo-daero, Yuseong-gu, Daejeon 34158, Korea; amoebatheo2009@gmail.com

**Keywords:** reactive sputtering, AlN, adhesion properties, thin-film encapsulation, crystallite size, parylene-C/AlN bilayer

## Abstract

Sputtering technique involves the use of plasma that locally heats surfaces of substrates during the deposition of atoms or molecules. This modifies the microstructure by increasing crystallinity and the adhesive properties of the substrate. In this study, the effect of sputtering on the microstructure of parylene-C was investigated in an aluminum nitride (AlN)-rich plasma environment. The sputtering process was carried out for 30, 45, 90 and 120 min on a 5 μm thick parylene-C film. Topography and morphology analyses were conducted on the parylene-C/AlN bilayers. Based on the experimental data, the results showed that the crystallinity of parylene-C/AlN bilayers was increased after 30 min of sputtering and remained saturated for 120 min. A scratch-resistance test conducted on the bilayers depicted that a higher force is required to delaminate the bilayers on top of the substrate. Thus, the adhesion properties of parylene-C/AlN bilayers were improved on glass substrate by about 17% during the variation of sputtering time.

## 1. Introduction

Parylene is a synthesized biocompatible polymeric coating material that is deposited on surfaces using the chemical vapor deposition (CVD) process developed by Gorham [1,2]. As a biocompatible and conformal coating polymeric material, parylene has several derivatives, which include parylene C, D, N, F, etc. [3]. The physical (transparent, opaque), chemical (corrosion and oxidation resistance), electrical (electrically insulative) and mechanical (flexibility and durability) properties of these materials have made them an ideal material that is applicable as a coating material on biomedical parts [2], transparent electronic devices such as organic light-emitting diodes (OLEDs) [4] and mechanical parts [5]. These properties are associated with the poly-crystalline nature, conformal coating, pinhole-free, low dielectric constant (~3), etc., of parylene materials [6]. Parylene films generally have poor adhesion to cold surfaces due to the lack of bonding between the surface and parylene film, a problem that is detrimental to surfaces coated with parylene for protection, biocompatibility, enhancing light extraction, etc. Over the years, this problem has been solved through either the application of adhesion promoters onto the substrate’s surface before parylene deposition and/or post-thermal treatment of parylene using plasma or annealing processes, to ensure strong adhesion [7,8,9]. The use of the plasma post-treatment technique on parylene has become more popular due to its ability to remove contaminating substances and tailor the wettability and adhesion properties of the surface [2,9]. Although this technique has already been used to improve the adhesion and other properties of parylene to surfaces, the technique is often applied to the surface of parylene film before it is attached to another surface. Tuning the crystallinity within parylene films plays a vital role in the performances of the films during application. For instance, altering the crystallinity improves the strength, mechanical modulus, fatigue resistance, chemical resistance, heat reflectance, increases the melting point, etc., of the parylene film [2]. This process is mainly achieved using the conventional post-annealing method to anneal the film at temperatures above its glass transition ($T_{g}$) but below its melting ($T_{m}$) temperature [10]. Research observations have shown that plasma from sputtering processes can also anneal (modify the crystallinity of) parylene films [11].

Sputtering is a physical vapor deposition (PVD) process that is relatively simple, directional and a satisfactory way of depositing thin films over flat surfaces [12]. During sputtering, ionized atoms or molecules generate plasma in a high vacuum chamber. Plasma is a well-known medium for the activation of surface bonds between materials. Additionally, it enables localized heating of the surface of the substrate, which can be used to modify the crystallinity of polymeric materials at the same or faster rate compared to the conventional annealing method [13]. The sputtering technique known as the reactive sputtering method is usually used in the production of thin-film encapsulation device which is made up of polymeric material and metal nitride or oxide (e.g., aluminum nitride-(AlN), silicon nitride-(${\mathrm{Si}}_{3}N_{4}$), aluminum oxide-(${\mathrm{Al}}_{2}O_{3}$), etc.), in a lamellae structure [14,15]. These metal nitrides or oxides are often used because of their low water vapor transmission rate (WVTR), high mechanical (high endurance to bending stress) and optical properties [15]. In its operation, nitrogen ($N_{2}$) or oxygen ($O_{2}$) gas and an argon (Ar) gas is simultaneously injected into a chamber, where the Ar atoms are ionized using radio frequency or a DC voltage to form a plasma [16]. The energized Ar ions accelerate and knock off atoms out of the surface of the target, which is usually an aluminum or silicon metal target. Since the process is characterized by an exothermic reaction, the atoms from the target react with $N_{2}$ or $O_{2}$ to form a group III nitride or oxide semiconductor, e.g., AlN, on the surface of the polymer. This results in the production of thin-film encapsulation layers with low WVTR and high endurance to bending stress [15].

Although the plasma generated in the sputtering process plays an important role during the film deposition, observations from subjecting parylene film to a range of temperatures during sputtering to form a bilayer can be beneficial in tuning the crystallinity and adhesion properties while ensuring a low WVTR and high endurance to bending stress of the film. This hypothesis, however, needs to be fully investigated to ascertain the effect of plasma on the microstructure of parylene as well as to propose the sputtering technique as an alternative way of modifying the adhesion properties of parylene on surfaces. Some research works over the years have been conducted, utilizing the sputtering method to fabricate a wide range of technological devices, for instance, semi-crystalline parylene to enhance the piezoelectric properties of flexible hybrid AlN materials [11], multilayered parylene-C/AlN used to enhance the shelf-life of OLED devices [17], multiple gas barrier layers fabricated using roll-to-roll sputtering [18], conformal hybrid piezoelectric-triboelectric ultra-thin wearable sensor made of AlN and parylene-C elastomeric blend, etc. [19]. However, these studies focused either on optimizing the device’s structure or developing new devices for their respective applications and did not take into consideration the effect of sputtering on the microstructure and adhesion properties of parylene film. In this study, the effect of plasma from sputtering on the microstructure and adhesive strength of parylene-C on a substrate was investigated using AlN in a reactive sputtering process without detaching the parylene film. The relationship between sputtering time and crystallinity, as well as the adhesion properties of the parylene-C/AlN bilayer on surfaces when subjected to sputtering processes, was also assessed. The results suggest that the plasma effect and the deposited molecules involved during reactive sputtering modified the crystallinity of the bilayer and, in effect, increased its adhesion properties on a glass substrate.

## 2. Materials and Methods

**Figure 1:**
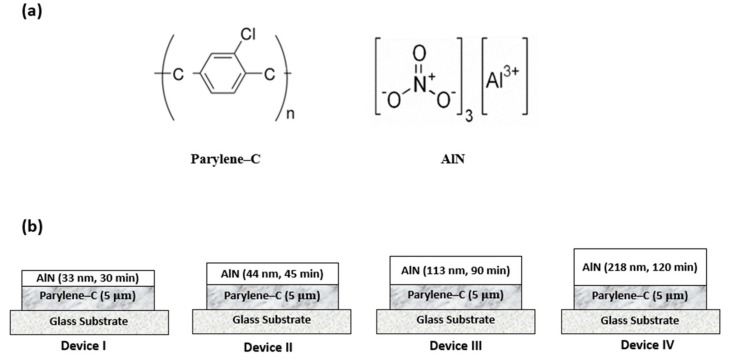
(**a**) The chemical structure of Parylene-C and AlN. (**b**) The schematic structures of AlN deposited on Parylene-C with respect to sputtering time.

Figure 1a shows the chemical structure of Parylene-C and AlN, respectively. Poly-chloro-para-xylylene (trade name: Parylene-C) was deposited using the Gorham chemical vapor deposition process that comprises of vaporization, pyrolysis, deposition and trapping processes [1]. Parylene-C was used due to its lower gas permeability and faster deposition rate compared to other derivatives. Parylene evaporator (EMBODYTECH, Daejeon, Korea) was used for the deposition process under high vacuum pressure (4 ± 0.5 mTorr). The parylene-C films were deposited on quartz and glass for optical analysis and structural characterization, respectively. In operation, the parylene powder (dimer: di-para-xylylene) was sublimed in the vaporization chamber, within a temperature step–range from 90–130 °C into the pyrolizer where the gaseous dimers split into monomers at 700 °C. The resulting monomeric gas then flew into the deposition chamber where it polymerized on the substrates at room temperature (25 °C) to achieve a transparent film. The thickness of the parylene-C film deposited was 5 μm.

Altering the crystallinity of Parylene-C has been predominantly demonstrated through conventional annealing [6,10]. After the parylene-C film was deposited on the substrates, the film on glass was annealed at 30, 60, 80 and 120 °C for 90 min and 80 °C, 90 °C each for 30, 45, 90 and 120 min. The annealing was done in the air on a hot plate at a rate of 5 °C/min and subsequently held for 30 min upon cooling. The annealing temperatures were purposely selected to compare the increasing crystallinity to that of the sputtering process.

AlN was then deposited on the surface of parylene-C, quartz and glass substrates using a reactive sputtering system. AlN was used due to its low water vapor transmission rate (WVTR) compared to other metal compounds. The deposition was done in a vacuum pressure (20 ± 3 mTorr), using a commercial Al target (Itasco) 99.999% of 100 mm in diameter. The sputtering process was carried out in a $N_{2}$/Ar gas-rich environment by varying the flow rate of $N_{2}$ and Ar gas at 5 ± 0.5 sccm and 18 ± 0.5 sccm, respectively, using a mass flow controller (MFC) (Nextron, Daejeon, Korea). A 300 W radio-frequency energy was applied using a radio-frequency (RF) power generator to form a controlled plasma during sputtering. Before the deposition of AlN on the various substrates, a pre-sputtering was conducted for 30 min to remove existing oxides on the surface of the target, eliminate oxygen within the chamber and stabilize the emanating plasma. The sputtering process was conducted for 30, 45, 90 and 120 min and AlN thickness of 33, 44, 113 and 218 nm were deposited on the substrates, respectively, as shown in Figure 1b.

The thickness of the various films was measured using the alpha step (Dektak-150, Veeco, Plainview, NY, USA). For optical transmittance analysis, the deposited films on quartz substrate were examined using an ultraviolet-visible-near-infrared (UV-vis-NIR) spectrophotometer (Lambda 950 UV-vis-NIR spectrophotometer, PerkinElmer, Waltham, MA, USA). The surface topography and morphology of the films were examined using a scanning electron microscope (SEM) (JSM-6390 (Jeol Ltd., Tokyo, Japan)) and Fourier transform infrared (FTIR) (Nicolet 6700 (Thermo, Waltham, MA, USA)), respectively. The traditional X-ray diffraction (XRD) θ–2θ scanning method using SmartLab (Rigaku, Tokyo, Japan) was used to characterize the crystallinity of the films.

For quantitative analyses, Scherrer’s formula [20] was used to calculate the crystallite size, τ, within the film:

(1) $$\tau =k\lambda /(\mathrm{BCos}\theta_{B})$$

where k is the shape factor ~0.9, λ is the wavelength of the X-ray beam: 1.5406 Å, B is the full width half maximum (FWHM) of the 2θ peaks in radian and $\theta_{B}$ is the Bragg’s angle. Finally, a mechanical scratch-resistance test was conducted on the films using Rockwell Nano-indentor and scratch tester (STeP4-NHT3) with a diamond tip of 10 μm to ascertain the adhesion properties of the films.

## 3. Results and Discussion

**Figure 2:**
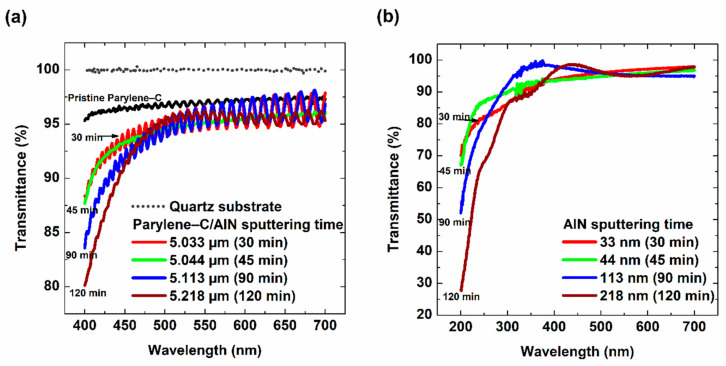
The transmittance spectra. (**a**) Quartz substrate (reference), pristine parylene-C and parylene-C/AlN samples, in the visible range. (**b**) Quartz substrate (reference) and AlN samples with respect to sputtering time.

Highly transparent parylene-C films are desirable in some applications such as light extraction in OLEDs, coating of micro-electromechanical systems (MEMS) and some biomedical parts. Figure 2 shows the optical analysis performed on the parylene-C/AlN bilayers and AlN films deposited on quartz to ascertain the transmittance of the film. Due to increased thickness with respect to the sputtering time of AlN films deposited on parylene-C and the quartz substrate, a decreasing trend in the transmittance was expected [18]. The parylene-C/AlN films showed a high transmittance of approximately 93% in the visible light range (from 400 to 700 nm), which is not widely deviated from the transmittance of the pristine parylene-C film, as depicted in Figure 2a. This indicates that, even though AlN of different thicknesses were deposited on the parylene-C, the transmittance of the bilayer was not significantly affected. Thus, the bilayer can be used for applications that require highly transparent parylene-C films. Among the films, AlN deposited on parylene-C for 120 min showed the least transmittance when compared to that of the pristine parylene. This could be due to the increased thickness and crystallinity within the bilayer with respect to sputtering time. The results correspond to the transmittance results of AlN samples deposited on quartz with respect to sputtering time, as shown in Figure 2b. The deposited AlN samples showed some absorptions only in the UV range and high transmittance near 95% with an oscillation behavior within the visible range. Kim et al. [18] reported similar observations on the decreasing transmittance of gas barrier films based on the increasing thickness of the films. The oscillations observed in the transmittance results are due to the waveguide of the thin film, which is dependent on the thickness of the films [21]. In contrast, the decreasing light transmittance within the visible range of the bilayers can be attributed to the thickness of the films.

**Figure 3:**
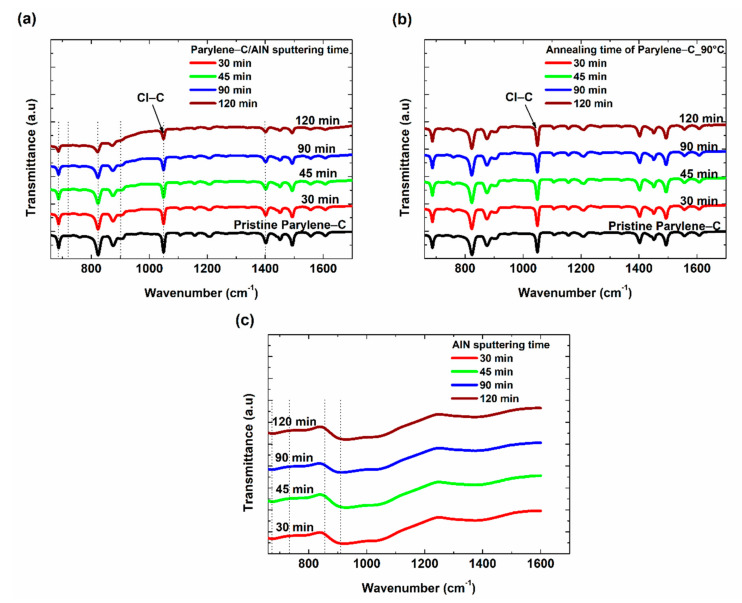
FTIR spectra. (**a**) Pristine parylene-C and parylene-C/AlN samples. (**b**) 90 °C Annealed parylene-C. (**c**) AlN samples, with respect to sputtering time.

FTIR measurements were conducted on the parylene-C/AlN bilayers, and the results were compared to that of the FTIR spectra of 80 °C and 90 °C annealed and pristine parylene-C films. FTIR uses infrared light to determine the chemical bonds and functional groups within a material at different wavelengths [4]. As the different chemical bonding groups absorb different wavelengths of infrared light, the FTIR spectrum gives specific information in the form of peaks relating to the bonds and functional groups present in the molecule of interest. The formation of new or disappearance of existing peaks within the measured spectrum, when compared to that of the standardized spectra, is a phenomenon that depicts the formation of new bonds or breaking of existing bonds within the molecule, respectively. This study was carried out in the absorbance mode within the wavenumber region of 600–1700 ${\mathrm{cm}}^{-1}$. Figure 3 presents the FTIR transmittance spectra of parylene-C/AlN, AlN films and annealed parylene (at 80 °C and 90 °C) films with respect to time. The peaks within the parylene-C/AlN and the annealed parylene spectra depict the major bonds within parylene-C, which include 1607 ${\mathrm{cm}}^{-1}$ (skeletal aromatic C-C vibrations), peaks at 1500, 1450 and 815 ${\mathrm{cm}}^{-1}$ (unsymmetrical trisubstituted benzene moiety), 1403 ${\mathrm{cm}}^{-1}$ (C-C strain bond), 1198 ${\mathrm{cm}}^{-1}$ (in-plane modification of C-H bond in the aromatic ring vibration), 1050 ${\mathrm{cm}}^{-1}$ (Cl-C bond of parylene-C), 876 ${\mathrm{cm}}^{-1}$ (single H bond to the ring with adjacent Cl and ethyl groups), 838 ${\mathrm{cm}}^{-1}$ (intramolecular C-H bond) and peaks at 709, 676 and 655 ${\mathrm{cm}}^{-1}$ (originated from amounts of parylene-C dimers) [4,22]. The FTIR spectra of AlN films deposited on glass exhibited a strong absorption near 900 ${\mathrm{cm}}^{-1}$, for all films with respect to sputtering time, as shown in Figure 3c. Alizadeh et al. [13] reported the FTIR transmittance spectrum of AlN on different substrates. The results suggest that AlN exhibits different absorption spectra on different substrates. Thus, the nonconforming FTIR spectrum of AlN to the standardized AlN wurtzite phase spectrum can be attributed to the different infrared absorption of AlN on the glass substrate. A general decrease in the absorption peaks was detected in the parylene-C/AlN spectra, as shown in Figure 3a. These decrements are neither due to the breaking of existing bonds nor the formation of new bonds but could be due to the increasing thickness of AlN, impeding the penetration of infrared through the films. This confirms the thickness of the AlN films deposited on parylene-C with respect to sputtering time, which is also consistent with the results depicted in the optical transmittance spectra. Similarly, the 80 °C and 90 °C annealed parylene-C spectra shown in Figure S1-Supplementary Material showed no new peaks or disappearance of existing peaks when compared to the spectrum of pristine parylene-C. These indicate that the sputtering processes the parylene-C was subjected to and the annealing temperatures were not sufficient enough to break or form any new bonds within the film [22], which can cause a change in color within the film. Note that the FTIR spectra of 80 °C and 90 °C annealed parylene-C films are similar, which is the reason for presenting only the spectra of 90 °C annealed parylene-C in Figure 3b.

**Figure 4:**
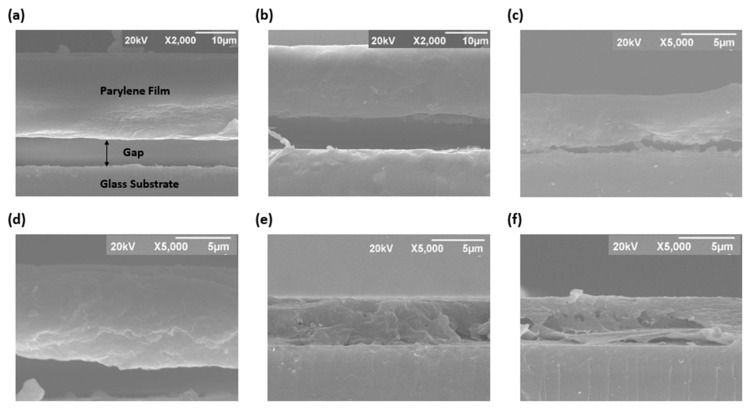
Cross-sectional SEM image. (**a**) Pristine parylene-C. (**b**) 90 °C annealed parylene-C for 120 min. (**c**) Parylene-C/AlN 30 min. (**d**) Parylene-C/AlN 45 min. (**e**) Parylene-C/AlN 90 min. (**f**) Parylene-C/AlN 120 min.

Structural characterization of the films was carried out using the scanning electron microscope (SEM). An SEM cross-sectional analysis was conducted using an operating voltage of 20 kV to evaluate the cross-section of the films. This was achieved by using a diamond tip to make a cross-sectional mark at the back of the glass substrate before the deposition of the films. The samples were then frozen using liquid nitrogen gas before being carefully broken into sections. Figure 4 shows the cross-sectional image of parylene-C/AlN bilayers compared to that of the pristine parylene-C and annealed parylene-C at 90 °C for 120 min. The pristine parylene-C showed a smooth texture film, similar to the annealed parylene-C film, indicating the dominating amorphous phase within the film [23]. Comparing the cross-sectional view of pristine parylene-C film to parylene-C/AlN bilayers, the parylene-C/AlN bilayers showed an increasing roughness in texture with respect to the sputtering time, depicting an increasing hardness of the bilayer. Moreover, an increasing flake on the cross-sectional view of the parylene-C/AlN bilayers was also observed. Similar roughness was reported in an AFM surface analysis by Gasonoo et al. [17] in an AlN reactive sputtering on parylene-C and Kahouli et al. [22] in the use of argon/hydrogen gas plasma (Ar/$H_{2}$) to treat the surface of parylene-C. These observations indicate an increasing crystallinity within the parylene-C/AlN bilayers subjected to Ar plasma and molecule deposition, hence the need for further investigations on the morphology of the films. Furthermore, a gap was observed between the film and glass substrate during the cross-sectional analysis, which could be due to the delamination of the film. The gap gradually diminished with respect to sputtering time from 30 to 120 min compared to that of the annealed and pristine parylene-C film. The diminishing gap in the parylene-C/AlN bilayer could be due to the increased deposition of AlN molecules and the plasma effect during sputtering.

**Figure 5:**
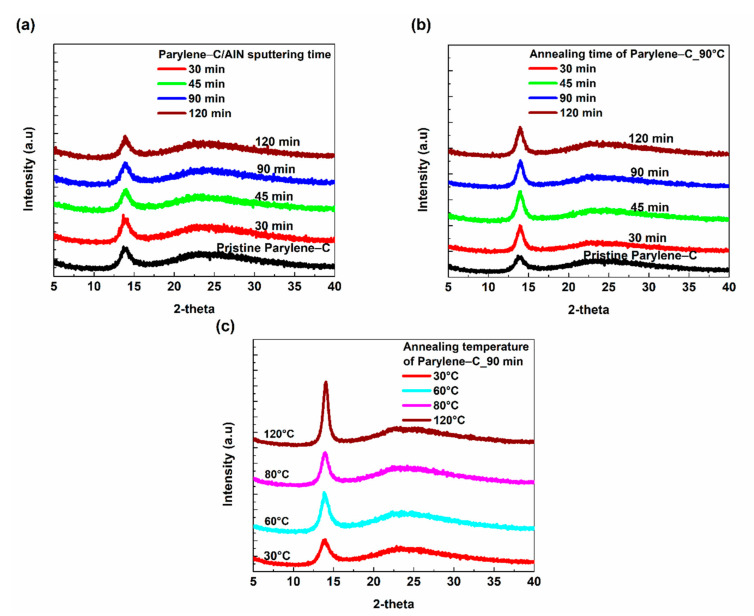
XRD patterns. (**a**) Pristine parylene-C and parylene-C/AlN samples. (**b**) 90 °C annealed parylene-C from 30–120 min. (**c**) 30, 60, 80 and 120 °C annealed parylene-C for 90 min.

The X-ray diffraction measurements were carried out to gain more insight into the effect of sputtering on the crystal structure of the parylene-C/AlN bilayer. The crystallinity of a polymeric material can be modified by subjecting the material to elevated temperatures and then allowing it to slowly cool to ambient temperature to assume a solid crystalline state [6,24]. The XRD results of parylene-C/AlN bilayers, annealed parylene-C at 90 °C for 30–120 min and annealed parylene-C at 30, 60, 80 and 120 °C for 90 min are shown in Figure 5. The results showed a sharp peak at 2θ = 14.0° and a broad peak around 2θ = 25° for parylene-C/AlN bilayers, annealed and pristine parylene-C films. These peaks correspond to the (020) reflection of the parylene-C monoclinic unit cell and amorphous phase within the crystalline-amorphous parylene-C, respectively [4,8]. The crystallinity of the parylene-C/AlN bilayers was compared to that of the annealed and pristine parylene films, with respect to sputtering and annealing time, respectively. An increment in the peak intensities of the parylene-C/AlN bilayers with respect to sputtering time, shown in Figure 5a, was observed compared to the pristine parylene-C, indicating an increasing crystallinity within the parylene-C/AlN bilayers. However, a significant increment in the peak intensities was observed for the annealed parylene-C samples compared to the pristine parylene-C and parylene-C/AlN bilayers, as shown in Figure 5b,c. The higher peak intensities could be due to the higher annealing temperatures compared to the emanating plasma temperature the parylene-C/AlN bilayer was subjected to. Golda-Cepa et al. [23] researched the effect of oxygen plasma on parylene-C, and Kaiser et al. [25] also researched the effect of hydrogen plasma on microcrystalline silicon. The group’s results showed that plasma can be used to modify the crystallinity of parylene-C and other polymeric materials. Jackson and Mathewson [11] also investigated the enhancement of piezoelectric properties of flexible hybrid AlN using semi-crystalline parylene, and the group observed that AlN deposition during sputtering caused the parylene substrate to be annealed (modified crystallinity). These reported results are in accordance with the increasing crystallinity within the parylene-C/AlN bilayers during the sputtering processes, as shown in Figure 5a.

**Figure 6:**
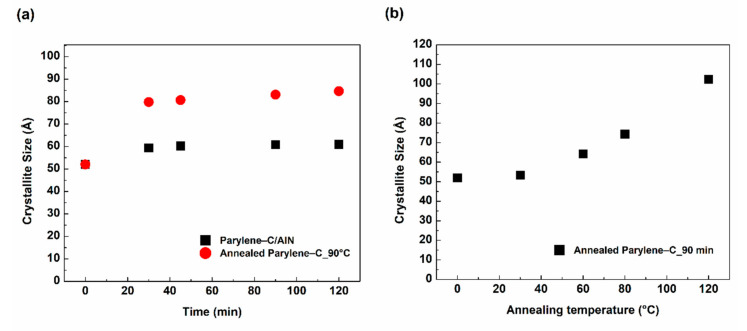
(**a**) Crystallite sizes of parylene-C/AlN and 90 °C annealed parylene-C with respect to sputtering and annealing time, respectively. (**b**) Crystallite sizes of 30, 60, 80 and 120 °C annealed parylene-C for 90 min.

To quantitatively examine the level of crystallization within the films, the crystallite size (τ) was calculated according to Scherrer’s equation (Equation (1)), and the results are presented in Figure 6. Pristine parylene-C recorded a crystallite size of 52 Å. The crystallite size of the parylene-C/AlN bilayers increased to 60 Å after 30 min of sputtering and was saturated to 120 min of the sputtering process. This is approximately a 16% increase in crystallite size above that of pristine parylene-C with respect to sputtering time. However, this crystallite size is relatively smaller when compared to that of the annealed parylene-C, which has an approximately 58% increase in crystallite size, as shown in Figure 6a, with respect to annealing time [6,8,10]. Based on the comparison of the parylene-C/AlN bilayers saturated results to annealed parylene-C at 30, 60, 80 and 120 °C for 90 min, it was clear that the sputtering process was conducted in a temperature environment which is equivalent to 60 °C annealed parylene-C film for 90 min, as shown in Figure 6b. This temperature falls within the glass-transition temperature (60–80 °C) of parylene-C [2]. Moreover, it reveals why the higher peak intensities (crystallinity) were observed in annealed parylene-C samples compared to parylene-C/AlN bilayers, which is due to higher annealing temperatures. The crystallite size results for both processes are presented in Table S1-Supplementary Material.

**Figure 7:**
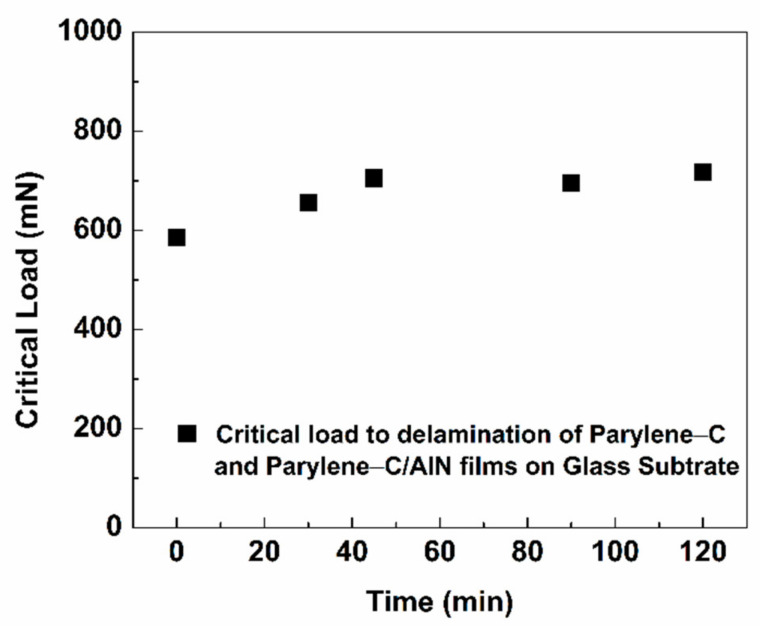
The critical load to delamination comparison of pristine parylene-C and parylene-C/AlN films, with respect to sputtering time.

Since the SEM results showed a diminishing delamination result of the parylene-C/AlN bilayers with respect to sputtering time, a scratch-resistant test measurement was conducted on the bilayers on a glass substrate to ascertain the adhesion properties of the films. The scratch test is one of the acceptable methods for measuring the adhesion properties of different coatings [7,26]. During its operation, a scratch is drawn across the surface of the film with a simultaneously constant or progressively applied load. At a certain critical point, the coating delaminates (viewed under an optical microscope), indicating the adhesion failure of the film. This point is called the critical load/force. Figure 7 presents the scratch-resistance test results of the parylene-C/AlN films on a glass substrate under an applied progressive force. The critical force at which the first delamination occurred is shown in the optical image (denoted as Lc1) of the parylene-C/AlN 90 min sample (scratch test results), shown in Figure S2-Supplementary Material. It has been observed that the parylene-C/AlN bilayers after 30 and 40 min of sputtering showed an increase in the critical load to delamination of the films by 17% on glass substrate above that of the pristine parylene-C, upon which it saturated to 120 min of sputtering. This essentially reveals that the effect of plasma and increasing thickness of AlN with respect to sputtering time modified the hardness and adhesion properties of the parylene-C/AlN bilayer on the substrate [27,28].

## 4. Conclusions

This study investigated the effect of sputtering on the microstructure and adhesive strength of parylene-C using AlN in a reactive sputtering process. The optical investigations on the parylene-C/AlN bilayers showed high transmittance of about 93%, which is suitable in the application of transparent devices. This revealed that AlN did not significantly affect the transmittance of parylene-C. The cross-sectional SEM results revealed that plasma and the deposited molecules through reactive sputtering onto the parylene-C film improve crystallinity and the hardness of the film. These results were supported by the XRD and scratch-resistance test results, respectively. The XRD results showed that the crystallinity of the parylene-C/AlN bilayers increased above that of the pristine parylene-C, within the saturated range from 30 to 120 min of the sputtering process. This reveals that plasma generated from sputtering can be used to modify the crystallinity of parylene-C/AlN bilayers. The scratch-resistant test results showed that the critical load to delamination of the bilayers increased by 17% above that of the pristine parylene-C. This reveals that the beneficial effects of sputtering on the crystallinity and adhesion properties of the bilayers are dependent on the time of exposure to plasma and the deposition of atoms or molecules during sputtering. Hence, the sputtering technique is an alternative technique that can be used to improve the adhesion properties of parylene films on substrates while enhancing the low WVTR and high endurance to bending stress of the film for electronic applications such as encapsulation and coating medical and mechanical devices for protection against corrosion.

## Data Availability

The data presented in this study are available within the article.

## Supplementary Materials

The following supporting information can be downloaded at: https://www.mdpi.com/article/10.3390/ma15155203/s1, Figure S1: FTIR spectra of 80 °C and 90 °C annealed parylene-C films at different annealing times. Both temperatures of annealed parylene-C showed same FTIR spectra as that of pristine parylene-C, indicating no breaking or formation of new bonds within the film; Figure S2: The scratch-resistant test was performed using Rockwell Nano-indentor and scratch tester (STeP4-NHT3) with a diamond tip of 10 μm. A progressive load was applied to the film. Presented is the scratch–resistance test result of parylene-C/AlN 90 min. In the optical image, Lc1 represents the critical force at which the first delamination of the film occurred. The various parameters, critical load, profile, acoustic emission, penetration depth and residual depth are indicated in the graph; Table S1: The XRD characteristics of parylene-C/AlN bilayers and 90 °C annealed parylene-C films with respect to sputtering and annealing time, respectively.
