# Supplementary material for: The Effect of Reactive Sputtering on the Microstructure of Parylene-C
Source: Materials (Basel). 2022 Jul 27;15(15):5203. doi: 10.3390/ma15155203 (PMC9369700; doi:10.3390/ma15155203)
Supplement: Supplementary file 1 [file materials-15-05203-s001.zip › materials-1811205-supplementary.pdf]

materials

Supplementary Materials

The Effect of Reactive Sputtering on the Microstructure of Parylene-C

Akeem Raji [1], Ye-Seul Lee [1], Seung-Yo Baek [1], Ji-Hyeon Yoon [1], Akpeko Gasonoo [2,†], Jonghee Lee [1] and Jae-Hyun Lee [1,*]

[1] Department of Creative Convergence Engineering, Hanbat National University, 125 Dongseo-daero, Yuseong-gu, Daejeon 34158, Korea; akeemraj61@gmail.com (A.R.); koko_42@naver.com (Y.-S.L.); hyo08177@naver.com (S.-Y.B.); jason7928@naver.com (J.-H.Y.); jonghee.lee@hanbat.ac.kr (J.L.)
[2] Research Institute of Printed Electronics & 3D Printing, Hanbat National University, 125 Dongseo-daero, Yuseong-gu, Daejeon 34158, Korea; amoebatheo2009@gmail.com
* Correspondence: jhyunlee@hanbat.ac.kr (J.-H.L.); Tel.: +82-(0)-42-821-1970
† Current address: Department of Chemistry, University of Calgary, 2500 University Drive N.W., Calgary, AB T2N 1N4, Canada.

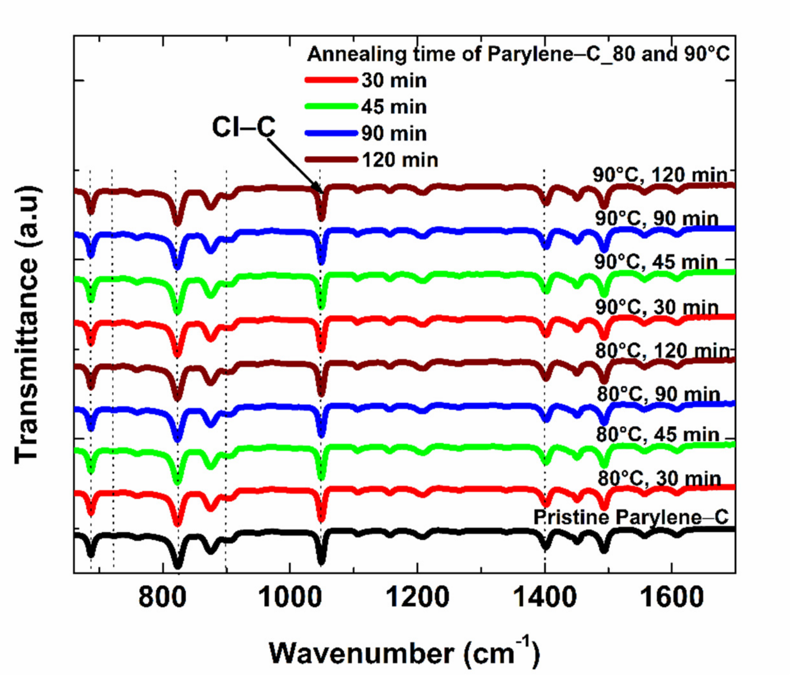

Figure S1. FTIR spectra of 80 °C and 90 °C annealed parylene-C films at different annealing times. Both temperatures of annealed parylene-C showed same FTIR spectra to that of pristine parylene-C indicating no breaking or formation of new bonds within the film.

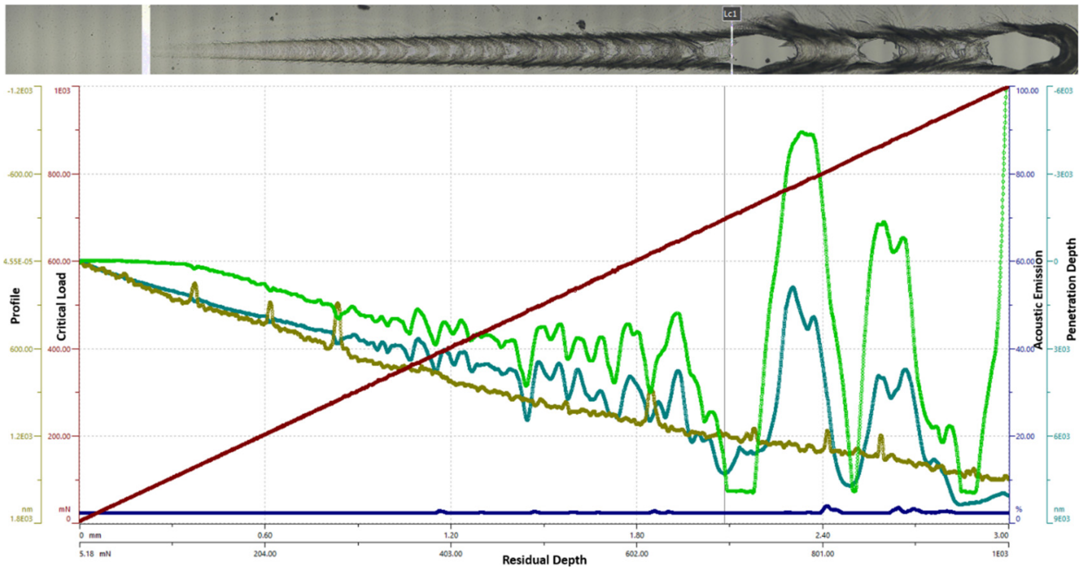

Figure S2. The scratch–resistant test was performed using Rockwell Nano–indentor & scratch tester (STeP4–NHT3) with a diamond tip of 10 μm. A progressive load was applied to the film. Presented is the scratch–resistance test result of parylene–C/AlN 90 minutes. In the optical image, Lc1 represents the critical force at which the first delamination of the film occurred. The various parameters, critical load, profile, acoustic emission, penetration depth and residual depth are indicated in the graph.

Table S1. The XRD characteristics of parylene-C/AlN bilayers and 90°C annealed parylene-C films with respect to sputtering and annealing time, respectively.

Time (min)	Samples	FWHM (2θ)	Crystallite Size (Å)	Crystallite Size (%)
0	Pristine Parylene–C	1.53	52	-
30	Parylene–C/AlN	1.34	59	13
	Ann Parylene–C_90°C	1.00	79	52
45	Parylene–C/AlN	1.33	60	15
	Ann Parylene–C_90°C	0.99	81	56
90	Parylene–C/AlN	1.32	61	17
	Ann Parylene–C_90°C	0.96	83	60
120	Parylene–C/AlN	1.31	62	19
	Ann Parylene–C_90°C	0.94	85	63
